# Human isolates of *Bartonella tamiae *induce pathology in experimentally inoculated immunocompetent mice

**DOI:** 10.1186/1471-2334-10-229

**Published:** 2010-07-30

**Authors:** Leah Colton, Nordin Zeidner, Tarah Lynch, Michael Y Kosoy

**Affiliations:** 1 Bacterial Diseases Branch, Division of Vector-Borne Diseases, Centers for Disease Control and Prevention, Fort Collins, CO, USA

## Abstract

**Background:**

*Bartonella tamiae*, a newly described bacterial species, was isolated from the blood of three hospitalized patients in Thailand. These patients presented with headache, myalgia, anemia, and mild liver function abnormalities. Since *B. tamiae *was presumed to be the cause of their illness, these isolates were inoculated into immunocompetent mice to determine their relative pathogenicity in inducing manifestations of disease and pathology similar to that observed in humans.

**Methods:**

Three groups of four Swiss Webster female mice aged 15-18 months were each inoculated with 10^6-7 ^colony forming units of one of three *B. tamiae *isolates [Th239, Th307, and Th339]. A mouse from each experimental group was sampled at 3, 4, 5 and 6 weeks post-inoculation. Two saline inoculated age-matched controls were included in the study. Samples collected at necropsy were evaluated for the presence of *B. tamiae *DNA, and tissues were formalin-fixed, stained with hematoxylin and eosin, and examined for histopathology.

**Results:**

Following inoculation with *B. tamiae*, mice developed ulcerative skin lesions and subcutaneous masses on the lateral thorax, as well as axillary and inguinal lymphadenopathy. *B. tamiae *DNA was found in subcutaneous masses, lymph node, and liver of inoculated mice. Histopathological changes were observed in tissues of inoculated mice, and severity of lesions correlated with the isolate inoculated, with the most severe pathology induced by *B. tamiae *Th239. Mice inoculated with Th239 and Th339 demonstrated myocarditis, lymphadenitis with associated vascular necrosis, and granulomatous hepatitis and nephritis with associated hepatocellular and renal necrosis. Mice inoculated with Th307 developed a deep dermatitis and granulomas within the kidneys.

**Conclusions:**

The three isolates of *B. tamiae *evaluated in this study induce disease in immunocompetent Swiss Webster mice up to 6 weeks after inoculation. The human patients from whom these isolates were obtained had clinical presentations consistent with the multi-organ pathology observed in mice in this study. This mouse model for *B. tamiae *induced disease not only strengthens the causal link between this pathogen and clinical illness in humans, but provides a model to further study the pathological processes induced by these bacteria.

## Background

*Bartonella *bacteria are small, fastidious, aerobic, Gram negative coccobacilli in the family *Bartonellaceae*, class Alphaproteobacteria. As of 2009 there are 24 named or proposed species in the genus [1], and over the last decade *Bartonella *bacteria have repeatedly emerged as a cause of human illness globally [2-6]. Infections with these bacteria are generally acquired from zoonotic sources such as host reservoir animals, or from hematophagous arthropods [1]. Either immunocompetent or immunocompromised people may become ill due to infections from *Bartonella *bacteria [7]. The two *Bartonella *species most commonly recognized as causing human illness are *Bartonella henselae*, the agent of 'cat scratch disease', and *B. quintana*, which caused numerous cases of trench fever in soldiers during World War I, and which today more typically infects homeless people, resulting in endocarditis or bacillary angiomatosis [8,9]. In South America, the lesser known though more virulent *Bartonella bacilliformis *causes Oroya fever and verruga peruana in humans, and infected patients may develop a severe, life-threatening hemolytic anemia [8].

Along with increasing awareness of *Bartonella *bacteria as emerging pathogens has come an increased interest in determining the mechanisms of pathogenesis of these microorganisms [10]. One of the primary impediments to our understanding of the infection and disease process elicited by these bacteria is the inability of researchers to establish suitable animal models for study [11]. Attempts to infect both immunocompetent and immunocompromised laboratory mice with various species of *Bartonella *bacteria have met with limited success [12-15]. *Bartonella *bacteria in these models tend to be quickly cleared by the immune system, and though the kinetics of antibody response and aspects of cellular and humoral immunity have been determined [16-18], animal models that reproduce clinical manifestations of disease observed in humans remain elusive. The natural reservoirs for *B. henselae *and *B. vinsonii *subsp. *berkhoffii *are cats and dogs (canines), respectively, and these animals can serve as models for *Bartonella *bacteria infection and disease processes [19-21]. However, though it may be more biologically appropriate at times to use natural hosts to study *Bartonella *bacteria induced pathogenesis, developing a mouse model for disease is desirable due to the widespread availability of murine immunological reagents and selective mutant strains of mice.

A recent success in developing an animal model for *Bartonella *induced disease was the inoculation of the human pathogen *B. henselae *into C57BL/6 and BALB/c mice, which produced lymphadenopathy of some duration [22]. These mice did not develop granulomas within the popliteal lymph node, and lymphadenopathy persisted in the near absence of detectable, viable bacteria, both in the lymph node and systemically [22], characteristics which also occur in human 'cat scratch disease', and which were reproduced in this mouse model of bartonellosis.

The present study was undertaken to establish the parameters for experimental inoculation of immunocompetent Swiss Webster mice, aged 15-18 months, with the suspected human pathogen *B.tamiae *[23]. Subcutaneous inoculation of three *B. tamiae *isolates into mice elicited a variety of severe histopathological changes and immune responses up to 6 weeks after inoculation. Our observations establish a murine model for *B.tamiae *induced disease and serve to implicate *B. tamiae *as a causative agent of human illness in Thailand.

## Methods

### Inoculation of mice

Specific pathogen free, outbred Swiss Webster female mice, aged 15-18 months, were used for this study. Mice were obtained from a colony of Swiss Webster mice maintained at the Centers for Disease Control and Prevention, Division of Vector-Borne Diseases (CDC/DVBD), Fort Collins, Colorado, USA, an Association for Assessment and Accreditation of Laboratory Animal Care (AAALAC) accredited facility. Work with the mice was approved by, and conducted under the supervision of the DVBD Institutional Animal Care and Use Committee [protocol # 07-012], in compliance with the guidelines set forth by the Animal Welfare Act (1966) of the United States of America, with regulatory oversight by the United States Public Health Service.

Bacteria for the mouse inoculations were grown on heart infusion agar plates supplemented with 10% rabbit blood [23], in a 5% CO_2 _incubator at 35°C. Bacterial colonies were harvested into phosphate buffered saline 5 days after plating, and stock suspensions were frozen at -80°C until thawed for inoculation. Aliquots of the frozen stock for each isolate were titrated to verify the inoculated dose. The *in vitro *bacterial passage history for each of the three *B. tamiae *isolates is detailed in Table 1[23,24], and does not include the preparation of the stocks described above.

**Table 1:** The *Bartonella tamiae *isolates used in the study have different *in vitro *bacterial passage histories

*B. tamiae *isolate	**Primary isolation from human blood samples, described previously in **[23]	Subsequent passage history (n = number of passages)
Th239	Blood clot from Thai patient inoculated onto Vero E6 cells	HIA supplemented with 5% rabbit blood, n = 6

Th307	Blood clot from Thai patient inoculated into BAPGM [24]	HIA supplemented with 5% rabbit blood, n = 2

Th339	Blood clot from Thai patient inoculated onto Vero E6 cells	BAPGM, n = 1; HIA supplemented with 5% rabbit blood, n = 2

HIA = Heart infusion agar plates

BAPGM = *Bartonella*/alpha-*Proteobacteria *growth medium [24]

All experimental mice were inoculated subcutaneously along the midline of the scruff of the neck with between 10^6 ^and 10^7 ^colony forming units of one of three *B. tamiae *isolates (Th239, Th307, or Th339) [23]. Three groups of four mice were chosen from the available pool of age matched female mice. Each group was randomly assigned to be inoculated with one of the three available *B. tamiae *isolates, such that four mice were inoculated for each of the three isolates [n = 12 experimental mice]. Two age-matched female mice were subcutaneously inoculated with saline to serve as controls. Experimental mice were group housed by isolate for the duration of the study, and control mice were held separately from experimental mice. One mouse per isolate group was sacrificed at 3, 4, 5, and 6 weeks post-inoculation (n = 1 mouse per timepoint for each isolate). All of the following tissues were sampled from each mouse, to include both experimental and control mice: blood, spleen, liver, lymph node(s), and kidney. Lymph nodes collected included axillary, brachial, inguinal, popliteal, and cervical nodes, generally in pairs. Lymph nodes were pooled for testing by PCR. Hearts were collected from mice during weeks 4 and 5 post-inoculation (n = 6 hearts total; 4 experimental and 2 control mice). Control mice were sacrificed at 4 and 5 weeks post-inoculation.

### Gross observations

Mice were examined by visual inspection and palpation in the weeks following inoculation. Development of ulcerations and formation of subcutaneous masses was noted, as were the location and number of skin lesions. Enlargement of axillary and inguinal lymph nodes was monitored by palpation, with reference to healthy, saline inoculated control mice.

### Polymerase chain reaction (PCR) detection of Bartonella DNA in tissues

Genomic DNA was extracted from tissues and blood sampled from Swiss Webster mice sacrificed at 3, 4, 5, and 6 weeks after inoculation with three *B.tamiae *isolates, and from saline inoculated control mice. A 200 ul sample of blood and 10-30 mg each of various tissues (lymph nodes, liver, spleen, kidney, and subcutaneous masses) from each mouse was subjected to DNA extraction using the manufacturer's blood or tissue protocol as outlined in the QIAamp DNA Mini kit handbook (Qiagen, Valencia, CA). Extracted DNA was stored at -20° C until tested.

*B. tamiae *DNA in samples was detected by PCR using *Bartonella *specific primers targeting the *gltA *(citrate synthase gene) [25], and 16S-23 S rRNA intergenic transcribed spacer (ITS) region [26] (Table 2). Polymerase chain reaction was performed in 50 μl reaction volumes containing extracted template DNA, 10 μl of 5× Green GoTaq reaction buffer, 200 μM of each dNTP, 0.5 μM of each forward and reverse primer and 1.25 U of Taq DNA polymerase (Promega, Madison, WI), in an Eppendorf Mastercycler (Eppendorf, Westbury, NY). The conditions for the *gltA *reactions were 2 min at 95°C, 35 cycles at 95°C for 30 s, 50°C for 45 s, 72°C for 30 s, and a 7 min extension at 72°C. The ITS PCR conditions were 2 min at 95°C followed by 40 cycles of 95°C for 30 s, 66°C for 1 min, 72°C for 1 min and a 7 min extension at 72°C. PCR products were visualized on a 1.5% agarose gel, and amplicons matching the target length were sequenced on a 3130 Genetic Analyzer (Applied Biosystems, Foster City, CA) to confirm their identity.

**Table 2:** *Bartonella tamiae *DNA in samples was detected by PCR using *Bartonella *specific primers

Primer name	Primer sequence 5'--3'	Amplicon size (bp)	Reference
gltA793F	CATGGTGGAGCTAATGAAG	344	This article

BhCS1137n	AATGCAAAAAGAACAGTAAACA		[25]

ITS325F	CTTCAGATGATGATCCCAAGCCTTCTGGCG	~300 bp	[26]

ITS1100R	GAACCGACGACCCCCTGCTTGCAAAGCA		

### Tissue preparation for histopathological analysis

Tissue samples were fixed in 10% neutral buffered formalin (Fisher Scientific, Kalamazoo, MI), subjected to standard processing, and embedded in paraffin. Sections of 5 μm were then prepared and stained with hematoxylin and eosin for evaluation by light microscopy (Colorado Histo-Prep, Fort Collins, Colorado, USA). Age-matched, saline inoculated control mouse tissues were treated in similar fashion, and all sections were read without prior knowledge of the experimental groups.

## Results

### Inoculation of mice--examinations and gross observations

Within the first week following inoculation, all mice exhibited thickened, tough skin at the inoculation site in the scruff of the neck. This resolved in surviving mice by week 4. The scruff skin of saline inoculated control mice remained thin and supple throughout the course of the study.

All mice inoculated with *B. tamiae *developed subcutaneous masses on the lateral thorax from 2 weeks post-inoculation, and some masses were still present at the conclusion of the study (6 weeks). Inguinal lymph node enlargement was detectable by palpation in mice during this same time period. Between weeks 2 and 3, mice inoculated with isolates Th307 and Th339 developed superficial ulcerations above several of the thoracic subcutaneous masses. Mice inoculated with Th239 did not develop skin ulcerations of subcutaneous masses. The subcutaneous masses did not appear painful upon palpation at any time during the course of the study, i.e. mice did not display aversion to handling or palpation of masses at any time.

### PCR detection of *Bartonella *DNA in tissues

Sequencing of PCR positive samples confirmed the presence of *B. tamiae *DNA in five mouse tissues (Table 3). *B. tamiae *DNA was detected 3 weeks post-inoculation in a subcutaneous mass and the liver of a mouse inoculated with Th339. DNA was also detected in the subcutaneous mass and lymph node of another mouse inoculated with Th339, 5 weeks post-inoculation. One mouse inoculated with Th307 was also found to have a *B. tamiae *DNA positive subcutaneous mass at week 3. No blood or tissue samples collected from mice inoculated with Th239 contained detectable *B. tamiae *DNA. Samples collected from the two saline inoculated control mice were not found to contain *B. tamiae *DNA by PCR analysis. Nucleotide sequence analysis indicated that the detected DNA was identical to the inoculated strains.

**Table 3:** *Bartonella tamiae *DNA was detected in tissues of inoculated mice by two specific PCR assays

Week post-inoculation	*B. tamiae *isolates	Tissue sample	*gltA*	ITS
3	Th339^a^	SQ mass	+	+

3	Th339^a^	Liver	-	+

3	Th307	SQ mass	+	+

5	Th339^b^	SQ mass	-	+

5	Th339^b^	Lymph node	+	+

^a ^the same mouse

^b ^the same mouse

SQ = subcutaneous

### Histopathological observations

As summarized in Table 4, differences in pathogenicity among *B. tamiae *isolates were noted. Th307 appeared less pathogenic than Th239 or Th339 when inoculated into mice, as only a deep dermatitis was seen at week 3, and a multifocal granulomatous nephritis was noted at weeks 5 and 6 after inoculation. This contrasts with Th239 and Th339 where granulomatous lesions were noted within the heart, kidney, liver and spleen of inoculated mice (Table 4). Lesions in the dermis occurred early after inoculation with Th307 and Th339 (3 weeks), while lesions of internal organs induced by all three isolates were noted at week 4 and persisted through week 6 post-inoculation (the duration of the study).

**Table 4:** Histopathological observations in aged, immunocompetent mice experimentally inoculated with three different *Bartonella tamiae *isolates [NAF = No Abnormal Finding]

*Bartonella tamiae *isolates				
**Week post-inoculation**		**Th239**	**Th307**	**Th339**

**3**	**Skin**	NAF	Dermatitis, deep, not oriented	Necrotizing dermatitis
	
	**Spleen**	NAF	NAF	NAF
	
	**Liver**	NAF	NAF	NAF

**4**	**Skin**	NAF	NAF	Necrotizing dermatitis
	
	**Spleen**	NAF	NAF	NAF
	
	**Liver**	Granulomatous hepatitis w/necrosis	NAF	Diffuse inflammatory response in sinusoids
	
	**Kidney**	Granulomatous nephritis	NAF	NAF

**5**	**Spleen**	NAF		Hemosiderosis in the cortex
	
	**Liver**	NAF	NAF	NAF
	
	**Kidney**	Perivascular granulomatous nephritis	Granulomas in the cortex and medulla	NAF
	
	**Lymph nodes**	Pyogranulomatous lymphadenitis	NAF	NAF
	
	**Heart**	Myocarditis, granulomas right & left atria, infiltrates	No sample	Myocarditis, granulomas right & left atria, infiltrates

**6**	**Skin**	No sample	Deep dermatitis	No sample
	
	**Spleen**	Pyogranulomatous nodules in the red and white pulp	NAF	NAF
	
	**Liver**	Hepatitis, granulomas w/necrosis	NAF	Perivascular granulomas, hepatocellular necrosis
	
	**Kidney**	NAF	Large perivascular granulomas	NAF
	
	**Lymph nodes**	Lymphadenitis	NAF	Lymphadenitis, cortical granulomas, necrotizing vasculitis

**Figure 1:**
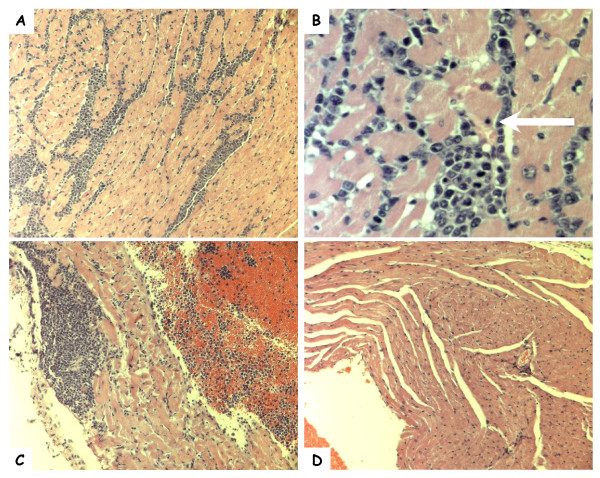
**Photomicrographs of hematoxylin and eosin stained heart sections of mice sampled during the study**. Mononuclear cell infiltration of the ventricle (A, 10×; B, 40×) and atrium (C, 10×) of a Swiss Webster mouse 5 weeks post-inoculation with *B. tamiae *Th339. The white arrow indicates a myocyte with a pyknotic nucleus. (D) Ventricle of a Swiss Webster mouse inoculated with saline (10×).

At week 5, in the hearts of mice inoculated with Th239 and Th339, a diffuse myocarditis was noted. Inflammation consisted primarily of mononuclear cells (lymphocytes and plasma cells) admixed with neutrophils occurring between myocytes (Figure 1-A). As noted in Figure 1-B, pyknotic nuclei and necrosis of adjacent myocardial muscle cells was seen (Figure 1-B, arrow). Also, granulomas were seen within both the right and left atria, with necrosis of adjacent muscle cells (Figure 1-C).

**Figure 2:**
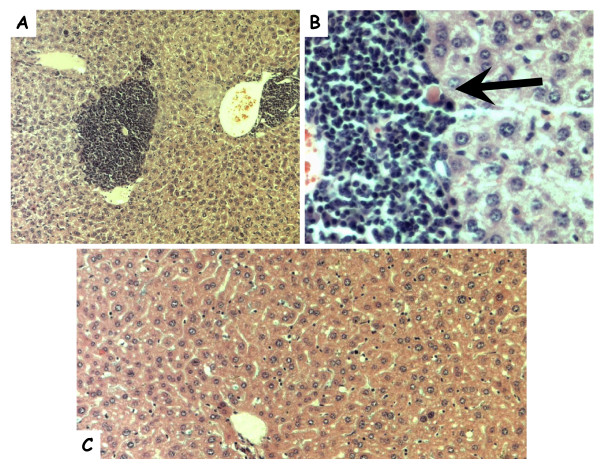
**Photomicrographs of hematoxylin and eosin stained liver sections from mice sampled during the study**. Granulomatous cell infiltration in liver tissue (A, 10×; B, 40×) of a Swiss Webster mouse inoculated with *B. tamiae *Th239. The black arrow indicates a necrotic hepatocyte. (C) Section of liver from a Swiss Webster mouse inoculated with saline (20×).

**Figure 3:**
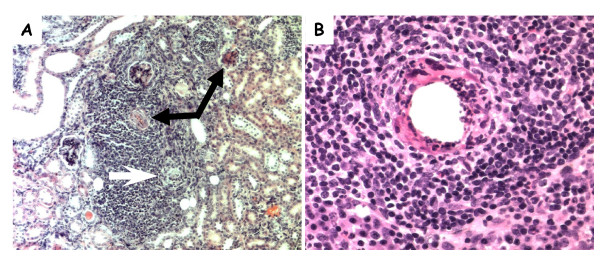
**Photomicrographs of hematoxylin and eosin stained kidney and lymph node sections from mice sampled during the study**. Granulomatous cell infiltration in the kidney (A, 10×) of a Swiss Webster mouse inoculated with *B. tamiae *Th239. Black arrows indicate degenerative glomeruli; the white arrow indicates a degenerating proximal tubule. (B) Necrotizing vasculitis in an axillary lymph node recovered from a Swiss Webster mouse 6 weeks after inoculation with *B. tamiae *Th339 (40×).

In the liver, multifocal pyogranulomatous infiltrates were noted adjacent to central veins (Figure 2-A) in association with hepatocellular necrosis (Figure 2-B, arrow). In the kidneys a perivascular granulomatous nephritis with associated degeneration and necrosis of glomeruli was noted in both kidneys (Figure 3-A, black arrows). These granulomas appeared to be associated with degeneration of proximal tubules (Figure 3-A, white arrow), and inflammation in kidneys was seen primarily within the renal cortex. Lesions within internal organs appeared to be perivascular, and, as noted in Figure 3-B, a necrotizing vasculitis occurred in prominent arteries of the cortex of the lymph nodes draining associated skin lesions. In the spleen, a necrotizing splenitis was seen, with a mixed inflammatory response occurring within both the red and white pulp (not shown). In the case of a mouse inoculated with Th339, a diffuse and prominent hemosiderosis was also seen within the white pulp of the spleen.

In the skin, caudal and lateral to the *B. tamiae *inoculation site, a necrotizing, ulcerative dermatitis developed dorsal to the subcutaneous masses early (3 weeks after inoculation of the mice with Th339, Table 4). The dermatitis persisted throughout the six week study period in some mice. Grossly, the lesions induced by Th339 appeared as ulcerative nodules, while microscopically, a severe, mixed inflammatory response was noted within the deeper layers of the dermis and subcutaneous tissues with associated necrosis of adjacent structures, such as hair follicles and sebaceous glands. Inflammatory infiltrates appeared similar to what was noted in the heart, liver, and kidney lesions, consisting of neutrophils admixed with mononuclear cells, predominantly plasma cells and mature lymphocytes. None of the above described lesions were seen in saline-inoculated control mice.

## Discussion

Inoculation of Swiss Webster mice aged 15-18 months with human isolates of *B. tamiae *induced disease processes consistent with clinical manifestations of disease observed in human patients [23]. Since mice experience age-related changes in immune function, which include alterations in T-cell responsiveness to antigens, this may have affected the outcome of our study [27,28]. Future studies will include younger mice to evaluate for age differences in response to this pathogen.

Two of the evaluated isolates of *B. tamiae*, Th239 and Th339, appear more pathogenic than the third, Th307 (Table 4). This corresponds to the presentation of the human patients from whom these isolates were obtained [23]. Patients 239 and 339 had rashes, and were febrile for 6 and 1 day(s) respectively, whereas Patient 307 was afebrile, and presented with pterygium in both eyes: all three patients had anemia [23]. In addition, the report of liver function abnormalities found in the human patients [23] would be consistent with hepatocellular disease, also seen in mice in the present study (Table 4, Figure 2). Immunopathological changes in the liver are not uncommon following human and cat infection with *B. henselae *[29-32], or *Bartonella clarridgeiae *[31]. *B. tamiae *DNA was found in the liver of one mouse infected with Th339 three weeks after inoculation (Table 3). Although *B.tamiae *DNA was detected, the question remains whether persistent bacteria in tissues or an inflammatory response directed toward bacterial antigen(s) was responsible for the perivascular granulomas and hepatocellular necrosis seen at week 6 in our mice (Table 4). In fact, it remains unclear whether any of the lesions observed were induced, and persisted or progressed in the presence or absence of viable bacteria.

It is unknown whether the three human patients infected with *B. tamiae *had cardiac disease [23]. No clinical tests were reported to have been conducted to evaluate for cardiac function abnormalities [23]. In the present study, *B. tamiae *Th239 and Th339 produced myocarditis in mice, with a diffuse inflammatory response associated with myocardial cell necrosis within both ventricles (Figure 1). Moreover, granulomatous lesions were observed in both atria 5 weeks post-inoculation (Table 4, Figure 1). Myocarditis in humans and animals has been associated with several *Bartonella *species [31,33-36]. Advances in diagnostic techniques have implicated *B. quintana*, *B. henselae*, and *B. elizabethae *in the majority of *Bartonella*-associated human myocarditis cases [37-39], while *B. vinsonii *subspp. *berkhoffii *seems to be the main cause of *Bartonella *induced myocarditis in dogs [33]. Histopathological findings in experimentally infected cats, and in naturally acquired human and dog cases of *Bartonella *myocarditis, are consistent with our observations in mice [31,33,40]. Shared characteristics of infection of heart tissue among these cases and our murine model include mixed inflammatory infiltrates [31,33,40], and myocyte necrosis [33,40], with random inflammatory foci found throughout heart tissue (Figure 1).

To our knowledge this is the first report of myocarditis associated with the inoculation of *B. tamiae*. Though the mice in our study had a diffuse myocarditis, and no endocarditis was found in hearts sampled during our study (n = 4; hearts sampled from experimentally inoculated mice at weeks 4 and 5), it is intriguing to note that a high rate of culture negative infective endocarditis exists in Khon Kaen, Thailand [41]. This is the same area where the patients reside from whom *B. tamiae *was isolated [23]. In the human endocarditis cases, the causative agent(s) is unknown, but the possible involvement of *Bartonella *bacteria has not been stringently evaluated [41]. In Thai patients with infective endocarditis, the mean period of time from symptoms to diagnosis was 5.7 weeks [41], and our present study only lasted to 6 weeks, with mouse hearts sampled at weeks 4 and 5 only. Further evaluation of our mouse model for *B. tamiae *induced disease may reveal more extensive cardiac involvement, especially in the context of a longer term study. Additional studies are also needed to determine the immunopathogenesis of these lesions in this murine model.

Lymphadenitis, and lymphadenitis with vasculitis, was seen in mice inoculated with *B. tamiae *Th239 and Th339, and sacrificed at weeks 5 and 6, and week 6 respectively (Table 4). This finding is consistent with presentations of *Bartonella *infections in human patients, as lymphadenitis is a common manifestation in immunocompetent humans infected with *B. henselae *[42]. It has also been observed in humans during infection with *B. quintana *[43] and *B. alsatica *[44], and in dogs [45] and cats infected with *B. henselae *[46]. Though lymphadenitis was observed microscopically, no *Bartonella *DNA was detected in those three lymph node samples. A recently published *B. henselae *'cat scratch disease' mouse model also reported persistent lymphadenopathy in mice, without detection of bacteria in the lymph nodes [22]. Conversely, in this study at week 5, *B. tamiae *DNA was detected by PCR in the lymph node of a mouse inoculated with Th339 (Table 3), but lymphadenitis was not observed in those tissues under microscopic examination. It appears that the presence of *Bartonella *DNA is not necessarily a predictor of pathological findings in the lymph nodes. Interestingly, of the four mice in our study inoculated with *B. tamiae *Th307, none displayed lymphadenitis, which supports our conclusion that this isolate differs in pathogenicity compared to strains Th239 and Th339.

Since little is known of the natural history of *B. tamiae *in Thailand, it is difficult to assess and quantitate human risk for acquiring infection with this suspected human pathogen [23]. Although a specific animal reservoir for the bacteria has not been identified, the epidemiological profile of the three patients shows some shared exposures congruent with *Bartonella *bacterial infections manifesting most often as zoonoses [23]. All three patients had a history of exposure to rats, and two had noted the appearance of rats in their homes in the weeks prior to the onset of their illness [23]. In recent years, a number of rodent associated *Bartonella *species have been isolated from patients exhibiting a wide variety of clinical illnesses [10]. These cases include *B. elizabethae*: endocarditis [10], *B. grahamii*: neuroretinitis [10], *B. washoensis*: myocarditis [10] and meningitis [4], and *B. vinsonii *subspp. *arupensis*: bacteremia with fever [10], and endocarditis [47]. To date, it remains unclear how these infections, as well as the human infections with *B. tamiae *[23], were acquired, whether by direct contact with an animal reservoir, or exposure to a hematophagous arthropod. Recently, *B. tamiae *DNA was detected by PCR assay in chigger mites and ticks collected from a variety of rodents in Thailand [48]. This finding suggests the involvement of chigger mites, ticks, and/or rodents in the transmission of *B. tamiae *to humans in Thailand.

## Conclusions

In this study we explored the capacity of three *B. tamiae *isolates to induce a variety of disease manifestations in aged immunocompetent mice. Thus far, our observations are consistent with the classification of *B. tamiae *as a human pathogen, since inoculation with the bacteria produced necrotizing dermatitis, lymphadenitis, granulomatous nephritis and hepatitis, and myocarditis in mice. A variety of disease characteristics observed in our mouse model correlate with what has been observed in other animal models and in human bartonellosis [31,33,40]. This murine model lends itself to the study of the immunopathogenesis of bartonellosis caused by *B. tamiae*, as it reproduces clinical symptomatology found in human patients in Thailand [23]. In addition, future studies in mice will evaluate the role age may play in the manifestation of disease. Finally, though the natural host and transmission dynamics of *B. tamiae *are unknown at this time, several lines of evidence suggest that rodents and/or ectoparasites can present some risk to humans for acquiring infection with this bacteria.

## Competing interests

The authors declare that they have no competing interests.

## Authors' contributions

MK and LC designed the study and performed the animal work. NZ assisted with the animal work, and made all the histopathological observations. TL carried out all testing of samples. LC drafted the manuscript, and it was reviewed by all other authors. All authors have read and approved the final manuscript.

## Pre-publication history

The pre-publication history for this paper can be accessed here:

http://www.biomedcentral.com/1471-2334/10/229/prepub
